# 2172. Activity of Cefiderocol and Comparator Agents Against Molecularly Characterized Multidrug-resistant Enterobacterales Clinical Isolates from United States Hospitals (2020–2022)

**DOI:** 10.1093/ofid/ofad500.1794

**Published:** 2023-11-27

**Authors:** Rodrigo E Mendes, John H Kimbrough, Valerie Kantro, Dee Shortridge, Helio S Sader, Mariana Castanheira

**Affiliations:** JMI Laboratories, North Liberty, Iowa; JMI Laboratories, North Liberty, Iowa; JMI Laboratories, North Liberty, Iowa; JMI Laboratories, North Liberty, Iowa; JMI Laboratories, North Liberty, Iowa; JMI Laboratories, North Liberty, Iowa

## Abstract

**Background:**

Cefiderocol (CFDC) is a siderophore cephalosporin that hijacks the iron transport system of Gram-negative bacteria to facilitate cell entry and reach its target. CFDC remains stable to hydrolysis in the presence of serine β-lactamases (ESBLs, KPC, and OXA-type carbapenemases) and metallo-β-lactamases (MBL). CFDC and comparator activities were analyzed against Enterobacterales (ENT), including molecularly characterized isolates, as part of the US SENTRY Antimicrobial Surveillance Program.

**Figure:**
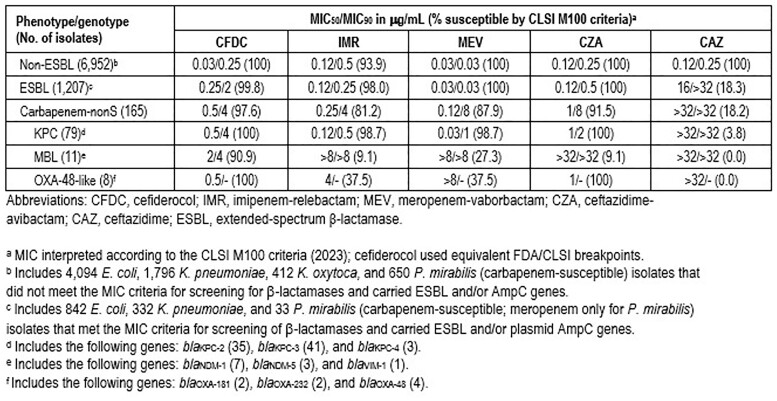

**Methods:**

11,884 ENT were collected from 33 sites in the USA in 2020–2022. Susceptibility (S) testing was performed by broth microdilution. CFDC testing utilized iron-depleted media. CLSI breakpoints were used. *E. coli*, *K. pneumoniae*, and *P. mirabilis* with ceftriaxone, ceftazidime, or aztreonam MIC ≥ 2 μg/mL plus any ENT displaying an MIC ≥ 2 μg/mL for imipenem (excluding *P. mirabilis, P. penneri*, and indole-positive Proteeae) or meropenem (MER) were subjected to genome sequencing and screening of β-lactamase genes.

**Results:**

CFDC (99.8%S), imipenem-relebactam (IMR; 98.0%S), meropenem-vaborbactam (MEV; 100%S), and ceftazidime-avibactam (CZA; 100%S) were active against carbapenem-susceptible ENT that carried ESBL and/or AmpC genes (Table). CFDC (MIC_50/90_, 0.5/4 μg/mL; 97.6%S) and CZA (MIC_50/90_, 1/8 μg/mL; 91.5%S) were the most active agents against carbapenem-nonS isolates, whereas IMR (MIC_50/90_, 0.25/4 μg/mL; 81.2%S) and MEV (MIC_50/90_, 0.12/8 μg/mL; 87.9%S) had suboptimal activity. CFDC (MIC_50/90_, 0.5/4 μg/mL), IMR (MIC_50/90_, 0.12/0.5 μg/mL), MEV (MIC_50/90_, 0.03/1 μg/mL), and CZA (MIC_50/90_, 1/2 μg/mL) were active (98.7–100%S) against the KPC subset. CFDC (MIC, 2/4 μg/mL; 90.9%S) was also active against ENT carrying MBL genes, whereas CFDC (MIC, 0.5-2 μg/mL; 100%S) and CZA (0.5-4 μg/mL; 100%S) were active against isolates carrying *bla*_OXA-48_–like.

**Conclusion:**

CFDC activity against ENT was consistent, regardless of isolate phenotypes or genotypes, including against isolates carrying carbapenemase genes other than *bla*_KPC_, where approved β-lactam/β-lactamase inhibitor combinations showed limited activity. These data emphasize CFDC as an important option for the treatment of infections caused by ENT and resistant subsets.

**Disclosures:**

**Rodrigo E. Mendes, PhD**, AbbVie: Grant/Research Support|Basilea: Grant/Research Support|Cipla: Grant/Research Support|Entasis: Grant/Research Support|GSK: Grant/Research Support|Paratek: Grant/Research Support|Pfizer: Grant/Research Support|Shionogi: Grant/Research Support **John H. Kimbrough, PhD**, AbbVie: Grant/Research Support|Basilea: Grant/Research Support|Pfizer: Grant/Research Support|Shionogi: Grant/Research Support **Valerie Kantro, BA**, AbbVie: Grant/Research Support|Pfizer: Grant/Research Support|Shionogi: Grant/Research Support **Dee Shortridge, PhD**, Melinta: Grant/Research Support|Shionogi: Grant/Research Support **Helio S. Sader, MD, PhD, FIDSA**, AbbVie: Grant/Research Support|Basilea: Grant/Research Support|Cipla: Grant/Research Support|Paratek: Grant/Research Support|Pfizer: Grant/Research Support|Shionogi: Grant/Research Support **Mariana Castanheira, PhD**, AbbVie: Grant/Research Support|Basilea: Grant/Research Support|bioMerieux: Grant/Research Support|Cipla: Grant/Research Support|CorMedix: Grant/Research Support|Entasis: Grant/Research Support|Melinta: Grant/Research Support|Paratek: Grant/Research Support|Pfizer: Grant/Research Support|Shionogi: Grant/Research Support

